# Attachment Styles and Sexual Function Among Survivors of Autologous Hematopoietic Stem Cell Transplantation: A Multicenter Study

**DOI:** 10.3390/medicina62010038

**Published:** 2025-12-24

**Authors:** Ioanna Tsatsou, Theocharis I. Konstantinidis, Kyriaki Mystakidou, Maria Nikoloudi, Eleni Panagou, Paraskevi-Maria Prapa, Maria Angelaki, Dimitra Bartzi, Ourania Govina

**Affiliations:** 1 One Day Clinic, Hellenic Airforce General Hospital, P. Kanellopoulou 3, 11525 Athens, Greece; itsatsou@uniwa.gr; 2 Nursing Department, Hellenic Mediterranean University, 71004 Herakleion, Greece; harriskon@hmu.gr; 3 Pain Relief and Palliative Care Unit, Department of Radiology, Areteion Hospital, School of Medicine, National & Kapodistrian University of Athens, Korinthias 27, 11526 Athens, Greece; mistakidou@yahoo.com (K.M.); mnikoloud@gmail.com (M.N.); 4 Nuclear Medicine Department, Hellenic Army General Hospital, P. Kanellopoulou, 11525 Athens, Greece; epanagou@uniwa.gr; 5 Oncology Department, Athens Hospital for Chest Diseases “Sotiria”, Mesogeion 152, 11527 Athens, Greece; eprapa@hotmail.com; 6 Psychiatric Department, Hellenic Airforce General Hospital, P. Kanellopoulou 3, 11523 Athens, Greece; aggelakimar@gmail.com; 7 Oncology Department, Hellenic Airforce General Hospital, P. Kanellopoulou 3, 11523 Athens, Greece; demmy345@hotmail.com; 8 Nursing Department, University of West Attica, Agiou Spiridonos 28, 12243 Athens, Greece

**Keywords:** autologous hematopoietic stem cell transplantation, survivors, sexual function, intimacy, relationships, attachment style

## Abstract

*Background and Objectives*: Autologous hematopoietic stem cell transplantation (AHSCT) offers life-saving treatment for hematologic malignancies but can result in persistent sexual dysfunction and relationship challenges. Attachment theory provides a valuable framework for understanding how enduring relational patterns influence sexual well-being. This study aimed to assess sexual function and attachment styles in AHSCT survivors and examine correlations between attachment and sexual health outcomes. *Materials and Methods*: A multicenter, cross-sectional study was conducted from December 2019 to March 2022 in five public hospitals in Athens, Greece. Participants were 127 adult survivors who had undergone AHSCT between 6 months and 5 years before enrollment. Sexual function was evaluated with the International Index of Erectile Function (IIEF) for men and the Female Sexual Function Index (FSFI) for women. Attachment style was measured using the Experience in Close Relationships Scale–Short Form (ECRSHORT-FORMSCALE). *Results*: Men demonstrated high erectile function (IIEF total: 54.10 ± 20.1), whereas women reported moderate sexual dysfunction (FSFI total: 22.51 ± 8.95). Both genders showed average attachment levels, with anxiety scoring lowest and discomfort with closeness highest. Between-group comparisons revealed no significant difference in anxiety (*p* = 0.95), a near-significant difference in avoidance (*p* = 0.056), and a significant difference in discomfort with closeness (*p* < 0.0001), with women scoring higher. In men, no significant correlations emerged between attachment and sexual function. In women, higher attachment anxiety correlated negatively with all FSFI domains except pain, avoidance correlated positively with lubrication, and discomfort with closeness correlated negatively with desire and pain. *Conclusions*: Findings reveal gender-specific patterns in attachment and their influence on sexual function of AHSCT survivors, highlighting the need for attachment-informed interventions to support intimacy, relationship satisfaction, and overall quality of life in survivorship care.

## 1. Introduction

Hematologic diseases and malignancies often require intensive treatment to restore healthy blood cell production. An effective therapeutic approach is hematopoietic stem cell transplantation (HSCT), which involves infusing healthy hematopoietic stem cells into patients. In autologous transplantation (AHSCT), especially for the treatment of lymphomas and multiple myeloma, these stem cells are collected from the patient themself and used to rebuild hemopoiesis after high-dose chemotherapy [1]. Providing supportive care to patients undergoing HSCT is challenging and complicated and plays a crucial role in influencing the treatment outcome [2].

Survivorship after HSCT refers to the phase in which patients live following the transplantation procedure, during which they face unique long-term medical, psychological, and social challenges despite the potential cure of their original disease. It encompasses managing late complications such as cardiac and vascular issues, secondary cancers, infectious diseases, and the effects on physical, psychological, and cognitive function [3]. Survivorship care aims to ensure early diagnosis, prevention, and management of these late effects while supporting quality of life and functional recovery over the long term. This period requires multidisciplinary follow-up and attention to both medical complications and psychosocial outcomes, including support for social reintegration and coping with functional limitations [4]. Compared with patients who have not undergone HSCT, those who have tend to have lower levels of quality of life (QOL). HSCT survivors often face a challenging recovery path marked by complex medical, social, and functional issues that collectively diminish their QOL [5].

Among the long-term consequences following HSCT, sexual dysfunction is consistently reported in QoL studies and constitutes a prevalent challenge [6]. The sexual side effects experienced by HSCT survivors are diverse, resulting from both the treatments and conditioning regimen and the subsequent supportive care drugs that impact both the body’s physical mechanisms and psychosocial well-being [7]. Physical symptoms like fatigue, pain, and insomnia, while biologically based, generate cognitive and emotional distress that directly hinder sexual activity and responsiveness. Sexual activity is also further impaired by psychosocial factors such as isolation, depression, distress, poor body image, and relationship and role changes [7,8]. The return to sexual activity after HSCT can also be influenced by the unrealistic expectation that responsiveness should immediately revert to pre-diagnosis levels. Meanwhile, a breakdown in communication, mutual understanding, and feelings of insecurity further diminish intimacy and prevent couples from successfully engaging in sexual intercourse after treatment [9,10].

Many relationship issues after HSCT can disrupt the sexual and psychological response of one or both partners. Changes in relationship balance, the patient’s intense preoccupation with their health, and the partner’s concern for the patient can potentially affect the couple’s intimacy and, consequently, their sexual interest and response to sexual messages or advances [11]. Couples or individuals who do not have children at the time of a transplant often experience concerns about infertility or sterility. Fertility concerns do not resolve over time and require many problem-solving discussions between couples [12]. Changes in roles due to the patient’s focus on survival, combined with the necessity for a spouse to take on a caregiver role, can hinder the return to sexual activity. Furthermore, partners may attempt to protect the patients from additional concerns and may not express sexual problems or concerns about fertility or intimacy issues, for fear of upsetting or burdening them [13,14].

Therefore, as HSCT becomes more common and patient survival increases, it is important to assess the quality of relationships of survivors after AHSCT, both from the physical context (sexual function) and the psychological one (relationship closeness). In this context, attachment theory offers a valuable framework for understanding how individuals navigate intimacy and emotional support after major medical interventions. The concept of adult attachment refers to the enduring expectations, needs, and preferences individuals hold regarding emotional closeness to significant others and the provision or receipt of support in times of need. Once established, attachment styles become relatively stable patterns that shape cognitions, emotions, and behaviors throughout the lifespan, influencing how individuals perceive relationships, regulate emotions, and respond to stress [15,16].

Rooted in Bowlby’s foundational work (1969) [17], attachment theory emphasizes the role of trust, verbal and nonverbal communication, intimacy, and protective caregiving, particularly when facing real or perceived threats to survival. In adulthood, these patterns are expressed most prominently within romantic and long-term partnerships, affecting not only relationship satisfaction but also broader aspects of psychological and sexual well-being [17]. Given its relevance, attachment theory has been widely applied in couple therapy, sexual counseling, and relationship interventions, where assessing an individual’s attachment style can clarify underlying dynamics, identify sources of conflict or disconnection, and guide the development of targeted, personalized treatment plans aimed at strengthening intimacy and resilience [18].

Based on this perspective, evaluating attachment styles in survivors of AHSCT can provide critical insights into the interplay between relational security, sexual function, and overall QOL. Such an approach can support clinicians in developing more holistic survivorship care strategies that address not only physical recovery but also the emotional and relational aspects essential to long-term well-being. Thus, the objective of this study was to assess the sexual function and quality of relationships among survivors of AHSCT, specifically investigating the connection between different attachment styles and sexual function outcomes.

## 2. Materials and Methods

### 2.1. Setting and Sample

A multicenter, observational, cross-sectional, and non-randomized study was carried out over a period from December 2019 to March 2022. The research was conducted at the hematology departments of five public hospitals in Athens, Greece, where participants were recruited through convenience sampling from survivors attending their outpatient follow-up after AHSCT. A total of 145AHSCT survivors were approached during the study period. Of these, 18 declined participation or did not meet eligibility criteria. The final study sample consisted of 127 participants, corresponding to a response rate of 87.6%. All enrolled participants completed the questionnaires and were included in the final analysis. Initial contact with each survivor was made in the presence of the attending physician or the responsible nurse. This approach facilitated reaching out to survivors and obtaining their consent, resulting in high response rates.

The study included participants who met the following criteria: they were adults over the age of 18, had not experienced a relapse in the past year, who had undergone AHSCT between 6 months and 5 years prior to the evaluation and had a functional status of 0–1 on the ECOG scale. A status of 0 indicated normal activity without limitations, as before the disease, while a status of 1 indicated some limitation in vigorous physical activities but preserved mobility and the ability to perform light work [19]. Participants also needed to be sexually active, defined as having engaged in sexual intercourse within the previous four weeks, possess satisfactory knowledge and understanding of the Greek language, and demonstrate adequate cognitive function, which encompasses abilities such as learning, thinking, reasoning, remembering, problem-solving, decision-making, and attention [20]. Survivors who had a diagnosed psychiatric disorder were excluded.

Although no specific statistical measures were applied a priori to control for biases, the study protocol incorporated procedural steps to ensure data reliability. Sampling bias was minimized through strict adherence to inclusion and exclusion criteria across five distinct study sites. To limit recall bias, validated instruments with a defined short-term recall period (four weeks) were used, and participants completed assessments on-site to prevent external influence.

### 2.2. Questionnaires

Survivors were asked to complete a questionnaire with demographic and clinical data, along with the Experience in Close Relationships Scale (ECRSHORT-FORMSCALE). For the assessment of sexual function, male survivors completed the International Index of Erectile Function (IIEF), while female survivors completed the Female Sexual Function Index (FSFI).

Clinical variables (including diagnosis, time since transplant, chemotherapy, and comorbidities) were extracted from the patients’ medical records at the time of study enrollment. Self-reported data (demographics, sexual function, and attachment style) were collected via paper-based questionnaires completed by participants in a private setting during their scheduled outpatient follow-up.

#### 2.2.1. Experience in Close Relationships Scale (ECRSHORT-FORMSCALE)

The ECRSHORT-FORMSCALE [21] is a modified version of the original 36-item scale (Experience in Close Relationships: ECR) that was created to measure the attachment of patients with advanced cancer to people in their environment [22]. It includes 16 questions classified into three subscales (attachment anxiety, avoidance, discomfort with closeness) that indicate three independent dimensions of the patients’ attachment style. Respondents are asked to rate each item on a Likert-type scale from 1 to 7 (1: strongly disagree, 7: strongly agree).

Attachment security is represented by lower scores as a securely attached person generally does not fear abandonment in a relationship (low anxiety) and is comfortable being dependent on others (low avoidance). It is a suitable scale for understanding coping mechanisms in medical settings, where health outcomes are influenced by individuals’ ability to seek out, trust, and interact with health professionals and their familiar environment [21]. Its assessment in the Greek language in cancer patients receiving palliative care as outpatients showed that it is reliable and valid, with psychometric properties similar to those reported in the international literature [23].

For the group of men survivors, the ECRSHORT-FORMSCALE Cronbach’s alpha values were 0.693 for “anxiety”, 0.624 for “avoidance” and 0.806 for “discomfort with closeness”. For women survivors, the ECRSHORT-FORMSCALE Cronbach’s alpha values were 0.798 for “anxiety”, 0.711 for “avoidance” and 0.831 for “discomfort with closeness”.

It should be noted here that, although the ECRSHORT-FORMSCALE is based on Likert-type items, its subscales represent composite scores with adequate internal consistency and were therefore treated as approximately continuous variables, consistent with established practice in psychometric and clinical research.

#### 2.2.2. International Index of Erectile Function (IIEF)

The IIEF is a validated 15-item tool used to measure erectile function based on the previous four weeks. It assesses five key areas: erectile function, orgasmic function, sexual desire, sexual satisfaction, and overall satisfaction. Each item requires a single response, typically rated on a scale of 0 (or 1) to 5, where lower cumulative scores suggest erectile dysfunction requiring further clinical investigation [24]. The IIEF was formally validated in Greek after undergoing the necessary cultural and linguistic modifications by Hatzimouratidis and colleagues [25].

The subscales of the IIEF exhibited outstanding internal consistency as measured by Cronbach’s alpha coefficient. The Cronbach’s alpha values were 0.986 for “erection”, 0.915 for “orgasm”, 0.979 for “desire”, 0.970 for “intercourse satisfaction”, and 0.962 for “overall satisfaction”.

#### 2.2.3. Female Sexual Function Index (FSFI)

The FSFI is a self-report questionnaire consisting of 19 items that evaluates six aspects of female sexual function: desire, arousal, lubrication, orgasm, satisfaction, and pain [26]. It has been validated for use among cancer survivors [27]. The FSFI uses questions pertaining to sexual function over the previous month, where items assessing frequency and intensity/response are scored on a five-point scale (with 0 for no sexual activity), and questions concerning pain or discomfort during or after vaginal penetration are reverse-coded so that higher scores denote less pain. The overall score of the FSFI is calculated by summing the results of the six subscales and is exclusively applicable to women who reported sexual activity in the previous month, where a greater score corresponds to superior female sexual function. Women whose total FSFI score falls below 26.5 are considered to potentially have some degree of sexual dysfunction requiring further clinical and laboratory examination [26]. The questionnaire has also been translated into Greek and formally validated for use in this cultural context by Zachariou and colleagues [28].

The subscales of the FSFI demonstrate excellent internal consistency, as indicated by the Cronbach’s alpha values (desire = 0.964, arousal = 0.974, lubrication = 0.984, orgasm = 0.973, satisfaction = 0.977, pain = 0.975, total score = 0.987).

### 2.3. Ethics

The study was carried out in compliance with the ethical guidelines set forth in the Declaration of Helsinki. The investigation was conducted following the acquisition of approval from the hospitals (protocol numbers: 1st hospital 656/14-11-2019, 2nd hospital 2775/4-2-20, 3rd hospital 1035/13-1-21, 4th hospital 4181/8-2-21, 5th hospital 1973/24-6-21) Ethics and Research Committees [15]. Before signing the informed consent form, participants were assured verbally and in writing regarding their anonymity, confidentiality, voluntary participation, and the right to withdraw at any time; they then completed the questionnaire during the same appointment in the researcher’s presence after receiving a full explanation, with the safeguarding of their personal information ensured through anonymous completion and the assignment of identification codes, while necessary clinical data were additionally gathered from medical records with the required permissions.

### 2.4. Statistical Analysis

Quantitative data were presented using the mean and standard deviation, while qualitative data were reported as frequencies and percentages. The Kolmogorov-Smirnov test was applied to check for the normality of the quantitative variables. To compare the ECR-SHORT FORMSCALE subscales between male and female survivors, an independent samples *t*-test was performed. Spearman’s correlation coefficients were used to explore the relationship between the sexual function scales and the ECR-SHORT FORM SCALE. Analyses were limited to descriptive statistics and bivariate associations, in line with the exploratory nature of the study. All statistical analyses were conducted using SPSS version 21.00 (IBM Corporation, Somers, Armonk, NY, USA), employing two-sided tests, with statistical significance defined by a *p*-value less than 0.05.

## 3. Results

### 3.1. Survivors’ Characteristics

The demographic and clinical characteristics of the 127 participants are summarized in Table 1. The majority of survivors were Greek men, married, employed, with children and graduates of higher education. The functional status score of the majority of survivors was ECOG 0, while they suffered from Hodgkin lymphoma. 54.4% of the patients had no recurrence of the disease, and all of them had received chemotherapy. After transplantation and treatments, 18.2% of the participants developed a musculoskeletal problem.

### 3.2. Descriptive Characteristics of the Measurement Scales

Based on established FSFI clinical cut-off values (FSFI score below 26.5 indicates some degree of sexual dysfunction), women AHSCT survivors demonstrated clinically relevant sexual dysfunction with a total score mean ± SD = 22.51 ± 8.96 (min–max: 1–36). Among the various domains of sexual function, the domain of “pain” recorded the highest score of 4.60 ± 1.54 (min–max: 0–6), while the domain of “orgasm” was the most adversely affected, scoring 3.19 ± 1.72 (min–max: 0–6) (Table 2).

Conversely, male survivors showed higher erectile function scores with a total mean score of mean ± SD = 54.07 ± 20.13 (min–max: 4–75), alongside lower overall sexual satisfaction [mean ± SD = 6.94 ± 2.47 (min–max: 2–10)] reflecting differential patterns across sexual function domains within this survivor cohort (Table 2).

Overall, both men and women had average values at the ECRSHORT-FORMSCALE subscales, indicating an average level of attachment in their close relationships. For men and women, the subscale of “anxiety” had the lowest values with mean ± SD = 13.48 ± 4.75 (min–max: 4–28) and mean ± SD = 12.89 ± 5.28 (min–max: 4–24), respectively. The subscale of “discomfort with closeness” had the highest values for both men and women survivors, with a mean ±SD = 19.90 ± 7.89 (min–max: 8–39) and mean ± SD = 34.48 ± 10.15 (min–max: 14–59), respectively.

Τhere was no significant gender difference for “anxiety”, a near-significant difference for “avoidance”, and a highly significant difference for “discomfort with closeness” of the ECRSHORT-FORMSCALE (Table 3).

### 3.3. Cοrrelatiοn οf Sexual Functiοn with the ECRSHORT-FORMSCALE

There were no statistically significant correlations between the subscales of the ECRSHORT-FORMSCALE and the subscales of the ΙΙΕF (Table 4).

Regarding the correlations between the FSFI and the ECRSHORT-FORMSCALE (Table 5), it was observed that “anxiety” presented a weak to moderate negative statistically significant correlation with all FSFI subscales except “pain”. This means that the higher the score on “anxiety” (which indicates that anxiety about the survivors’ attachment to their relationships increases), the lower the sexual functioning of women across all FSFI subscales, except for pain.

“Avoidance” presented a moderate positive statistically significant correlation with “lubrication” of the FSFI. This suggests that the higher the score on “avoidance” (which indicates that avoidance about the patients’ attachment to their relationships increases), the higher the score on the “lubrication” subscale of the FSFI, and therefore better functioning in this area.

Also, “discomfort with closeness” showed a weak, negative, statistically significant correlation with the “desire” and “pain” FSFI subscales. This indicates that the higher the score on “discomfort with closeness” (which suggests that discomfort with closeness increases in relation to patients’ attachment to their relationships), the lower the patients’ sexual functioning on the “desire” and “pain” subscales of the FSFI.

## 4. Discussion

In summary, the present study revealed distinct gender-specific patterns in both sexual function and attachment styles among AHSCT survivors. Men survivors demonstrated preserved erectile function but reported no significant correlation between attachment style and sexual outcomes. In contrast, women survivors exhibited high rates of sexual dysfunction and significantly higher levels of ‘discomfort with closeness’ compared to men. Furthermore, for women, attachment anxiety was found to be a significant negative correlate of sexual function, suggesting that relational insecurity impacts female sexual health more directly than male sexual health in this population.

Attachment theory has been described as one of the most influential theories for integrating the wide variety of coping mechanisms in cancer. Attachment is divided into three styles: secure, anxious and avoidant attachment. Attachment style also influences the receipt of care in the context of a life-threatening illness, such as cancer [29]. In cancer, avoidant individuals in their relationships withdraw from others in response to their distress, in line with their need for independence, regulating their negative emotions, and concealing their weaknesses [30]. They also tend to suppress the need to seek closeness, maintain an emotional distance from others, and react more negatively to receiving care, which can lead to significant distress in their relationship [31]. In contrast, anxious individuals try to draw others even closer, aligned with their fear of abandonment, have a strong need for support and affection, and may display intense emotions. Furthermore, anxious attachment usually leads to excessive use of maladaptive coping strategies, resulting in negative moods [23].

Attachment theories have been extended to understand adult romantic relationships and especially the role of sexuality within intimate relations [32]. Individuals who exhibit high attachment security are generally more comfortable with sexual intimacy, expressing more positive views and emotions regarding sexual activity, which research has linked to greater sexual satisfaction and reduced levels of sexual dysfunction when compared to those with attachment insecurity [33,34]. On the contrary, individuals characterized by high attachment anxiety tend to exhibit an obsessive focus on their romantic partners and often cling to them, resulting in more negative sexual thoughts and emotions and a perception of themselves as less sexually attractive than their securely attached counterparts [33,35].

The romantic relationships of individuals who exhibit high attachment avoidance are typically defined by emotional distance, a pervasive fear of intimacy, and resulting low levels of both trust and relationship satisfaction. These individuals dislike physical affection (kissing, hugging), and they experience more negative emotions in response to sexual activity [32]. Insecure attachment plays a key role in individuals with sexual dysfunctions [36]. Increased levels of both attachment anxiety and avoidance are linked to less satisfying sexual relationships, a greater prevalence of sexual dysfunction, and variations in both the frequency of sexual intercourse and the underlying motivations for engaging in it [34].

According to our findings, women survivors had an average level of attachment in the relationship. Higher anxiety and higher attachment avoidance were significantly and positively associated with anxiety and perceived negative impact of cancer in breast cancer patients [37]. The positive association between an insecure attachment style and poor psychological adjustment (e.g., psychological distress, poor QοL) has been found in breast cancer [37], lung cancer [38], and patients with metastatic cancer [39]. Patients who demonstrated high levels of both attachment anxiety and avoidance reported significantly greater levels of anxiety and depression alongside poorer quality in their marital relationships [37,38,39].

Sexual function, vaginal problems and avoidance directly impacted sexual satisfaction in a study of cervical cancer survivors. Marital adjustment was a partial mediator in the relationship between avoidance and sexual satisfaction. Additionally, both avoidance and anxious attachment affected sexual satisfaction indirectly through the mediation of marital adjustment. These findings imply that the deterioration of sexual function and satisfaction is not limited to physical dimensions but also is influenced by attachment style and marital adjustment [40]. Furthermore, a systematic review revealed that a more insecure attachment style leads to worse outcomes for patients regarding their psychological adjustment to cancer and their capacity to perceive and access available social support. On the other hand, a secure attachment style is linked to positive growth and enhanced well-being across all areas of an individual’s QoL [41].

Therefore, we could attribute our findings to the fact that women with insecurities in their relationships, anxiety, sadness, and distress are very likely to have affected sexual function as a correlation has been found between psychological and sexual problems [42], the security of attachment style and the general well-being of patients [41], and between the security of attachment style and the sexuality of individuals [32]. However, to attribute a more correct etiology, the context of the partner relationship and the partner should also be examined.

In our study, men survivors reported mean ECRSHORT-FORMSCALE scores indicative of a moderate level of attachment, with the lowest mean values in “anxiety” and the highest in “discomfort with closeness”. Interestingly, when comparing the two genders, no significant difference was observed in attachment-related anxiety, suggesting that concerns about abandonment or insecurity in relationships were comparable between men and women following AHSCT. This is in line with previous research in cancer survivors, indicating that attachment anxiety may be more strongly associated with personality traits and coping style rather than gender alone [37,38,39].

The difference in “avoidance” approached statistical significance, with men tending toward higher avoidance scores than women. This pattern aligns with the broader literature showing that men often exhibit more avoidant attachment strategies in the context of chronic illness, potentially as a coping mechanism to preserve autonomy and emotional control [30,43]. Such avoidant tendencies may influence both the willingness to seek intimacy and the openness to discuss emotional or sexual concerns after HSCT.

The most evident gender difference emerged in “discomfort with closeness”, where women scored significantly higher than men. A strong discomfort with closeness or intimacy and dependence in relationships is often expressed through emotional detachment, heightened self-sufficiency, and hesitation to disclose personal feelings or lean on others. People with high avoidance typically uphold strict emotional boundaries and may use protective strategies to guard against perceived risks in relationships [44]. This finding may reflect the complex psychosocial aftermath of HSCT for women, including altered body image, hormonal changes, and physical symptoms that affect sexual function and comfort with intimacy [6,32,45]. Women survivors may thus experience greater relational strain when physical closeness triggers vulnerability or reminders of illness.

When examining the correlations between sexual function and attachment, men’s IIEF scores showed no statistically significant association with any ECRSHORT-FORMSCALE subscale, suggesting that for male survivors in our sample, sexual function was not directly linked to attachment style. This might indicate that erectile function and related sexual outcomes are influenced more strongly by physiological factors (e.g., treatment side effects, comorbidities) rather than by relational attachment dimensions in the male survivor population [46].

In contrast, in women, higher attachment anxiety correlated moderately and negatively with nearly all FSFI domains except “pain”, implying that greater relational insecurity was associated with poorer sexual function in desire, arousal, lubrication, orgasm, and satisfaction. This aligns with previous findings in oncology populations, where attachment anxiety has been linked to heightened sexual distress, lower sexual satisfaction, and maladaptive coping with illness-related changes in sexuality [34,47,48]. The observed weak negative association between “discomfort with closeness” and FSFI “desire” and “pain” domains further suggests that physical intimacy discomfort may diminish sexual interest and exacerbate perceived pain during sexual activity, a pattern also described in gynecologic cancer survivors [40].

An unexpected finding was the moderate positive correlation between attachment avoidance and lubrication in women. Although counterintuitive, it may be that women with higher avoidance report lubrication in a more functional, less emotionally engaged context, reflecting a behavioral rather than emotional participation in sexual activity. Similar associations have been noted in the general population, where avoidance is linked to lower sexual scores but not necessarily to all physical aspects of sexual response [49].

Finally, for survivors and partners of allogenic HSCT, one recent study evaluated a psychosexual intervention and used the ECRSHORT-FORMSCALE before the intervention. The scores on the “anxiety” and “avoidance” subscales were higher than those that we found for the survivors of autologous HSCT. All five couples that participated expressed strong agreement or agreement that the intervention has facilitated a better understanding of the issues within their relationship that have impacted their sexual wellbeing as a couple [50].

Although no studies were found in the literature to assess these relationships specifically for survivors of AHSCT, the results are supported from the existing literature that demonstrates the significant influence of close relationships on an individual’s sexual function. Attachment theory offers a valuable framework for comprehending the individual differences observed in survivors’ intimacy and sexual recovery following transplantation, emphasizing that both physical and psychological rehabilitation are essential components of survivorship care [51].

### Limitations and Strengths

The generalizability of the study’s findings is limited by several limitations. Its cross-sectional design does not clearly establish the temporal relationship between cause and effect, although this type of study is common in this field. Additionally, the non-random convenience sampling limits the conclusions that can be drawn for the entire survivor of the AHSCT population. So, the influence of other factors on sexual dysfunction cannot be excluded. Sexual function is a multifactorial outcome influenced by biological, treatment-related, and psychosocial variables. The scope of the present study did not allow for multivariable modeling to examine the relative contribution of these factors.

To note, no a priori sample size or power calculation was performed, as the study was exploratory in nature and the sample size was determined by the availability of eligible AHSCT survivors during the study period. The small sample size, despite the multicenter approach, is a result of the specificity of the group, the restrictions posed by the COVID-19 pandemic (the period in which the study was conducted) and the strict participation criteria. The sensitive nature of the questions also led some patients to decline participation. Moreover, the study relied on self-reported questionnaires, with the limitations of subjective assessment, and did not include partners in the evaluation.

Furthermore, the absence of an age-, sex-, and partner-status–matched healthy control group limits conclusions regarding how sexual function in AHSCT survivors compares to the general population. Therefore, findings should be interpreted as clinically meaningful patterns within this survivor sample, based on validated instrument thresholds, rather than as relative impairment or preservation compared to healthy controls.

This study is the first multicenter investigation in Greece to examine both sexual function and attachment style in survivors of AHSCT, integrating psychosexual and relational frameworks within the context of long-term survivorship. By applying attachment theory to a post-transplant population, this research moves beyond the traditional biomedical focus of HSCT follow-up, offering new insights into how relational insecurities, specifically anxiety, avoidance, and discomfort with closeness, interact with sexual health outcomes. The gender-based comparative analysis revealed distinctive patterns, with no significant difference in anxiety, a near-significant difference in avoidance, and a pronounced difference in discomfort with closeness, while the correlation findings highlighted that women’s sexual function was more strongly associated with attachment dimensions than men’s. These findings underscore the importance of integrating attachment-informed assessment and counseling into survivorship care, contributing novel evidence that can guide both clinical practice and future research on the psychosocial rehabilitation of HSCT recipients.

## 5. Conclusions

Overall, these results underscore the complex interplay between psychosocial attachment dimensions and sexual health following AHSCT. Attachment patterns, formed early in life, significantly influence adult romantic and sexual relationships [52]. This multicenter research assessed and correlated the sexual function and attachment in close relationships among survivors of AHSCT. Notably, this is the first study conducted that acknowledges the influence of attachment in close relationships on sexual function within this particular group of survivors. By adopting this approach, the study tried to provide a more comprehensive understanding of the psychosocial and relational challenges faced by survivors, thereby informing targeted interventions to improve intimacy, relationship satisfaction, and overall quality of life.

Future studies could adopt a prospective design and include partners as well as members of sexual and gender minorities. They could also explore interventions targeting sexual dysfunction, emotional distress, and problematic relationship dynamics. Using a combination of quantitative and qualitative methods would also provide a deeper understanding of these topics. Εven though HSCT is a life-saving treatment fοr a number οf patients with hematolοgic malignancies, there is a notable incidence οf sexual dysfunction and emotional distress, with a subsequent impact on relationships and quality of life in the short and long term. This study tried to shed light on and raise awareness regarding the persistent psychosexual challenges faced by individuals who have undergone AHSCT. While these findings necessitate further validation with more research before they can be broadly applied, they offer useful information for guiding subsequent research.

## Figures and Tables

**Table 1:** Sociodemographic and clinical profile of the study participants (N = 127).

Variable	Categories	N (%)
Gender	Men	71 (55.9)
	Women	56 (44.1)
Age (years)	Mean ± SD	45.6 ± 12.8
Time since AHSCT (years)	Mean ± SD	3.1 ± 1.5
Functional Status (ECOG)	0 (Normal activity)	100 (78.7)
	1 (Restricted strenuous activity)	27 (21.3)
Marital Status	Married/Committed relationship	125 (97.2)
	Divorced/Single	2 (2.8)
Education Level	Basic/Secondary Education	59 (38.6)
	Higher/Post-graduate Education	68 (53.4)
Employment	Employed	75 (59.1)
	Unemployed/Retired	52 (40.9)
Primary Diagnosis	Hodgkin Lymphoma (HL)	60 (47.8)
	Non-Hodgkin Lymphoma (NHL)	35 (27.8)
	Multiple Myeloma (MM)	32 (24.4)
History of Relapse	No	69 (54.4)
	Yes	58 (45.6)
Additional Therapies	Radiotherapy	40 (31.5)
	Immunotherapy	40 (31.5)
	Targeted Therapies	17 (13.8)

ECOG: Eastern Cooperative Oncology Group performance status; AHSCT: Autologous Hematopoietic Stem Cell Transplantation; SD: standard deviation, NHL: Non-Hodgkin Lymphoma, HL: Hodgkin Lymphoma, MM: Multiple Myeloma.

**Table 2:** Descriptive statistics of sexual function (FSFI & IIEF) in AHSCT survivors.

Gender	Sexual Function Domain	Mean	SD	Range (Min–Max)
Women (FSFI)	Desire	3.42	1.35	1–6
	Arousal	3.50	1.63	0–6
	Lubrication	3.81	1.78	0–6
	Orgasm	3.19	1.72	0–6
	Satisfaction	3.99	1.70	0–6
	Pain	4.60	1.54	0–6
	FSFI Total Score	22.51	8.96	1–36
Men (IIEF)	Erectile Function	22.54	8.70	1–30
	Orgasm	7.87	2.98	0–10
	Sexual Desire	7.20	2.74	1–10
	Intercourse Satisfaction	9.52	3.90	0–15
	Overall Satisfaction	6.94	2.47	2–10
	IIEF Total Score	54.07	20.13	4–75

FSFI: Female Sexual Function Index; IIEF: International Index of Erectile Function; SD: Standard Deviation.

**Table 3:** ECRSHORT-FORMSCALE scores.

ECRSHORT-FORMSCALE	Gender	N	Mean ± SD	*p* *
Anxiety	Men	71	13.48 ± 4.75	0.95
	Women	56	12.89 ± 5.28	
Avoidance	Men	71	18.82 ± 4.66	0.056
	Women	56	17.02 ± 3.31	
Discomfort with closeness	Men	71	19.90 ± 7.89	<0.0001
	Women	56	34.48 ± 10.15	

ECRSHORT-FORMSCALE: Experience in Close Relationships Scale; SD: standard deviation. * independent samples *t*-test.

**Table 4:** Correlation between IIEF and ECRSHORT-FORMSCALE.

ΙΙΕF		Εrection	Orgasm	Desire	Ιntercourse Satisfaction	Overall Satisfaction	Τotal Score
ECRSHORT-FORMSCALE	Anxiety	0.008	−0.048	−0.042	−0.040	0.055	0.008
	Avoidance	0.092	0.117	0.097	0.091	0.009	0.092
	Discomfort with closeness	0.055	−0.019	0.049	0.016	0.129	0.055

IIEF: International Index of Erectile Function; ECRSHORT-FORMSCALE: Experience in Close Relationships Scale. All indices report Spearman’s correlation coefficient.

**Table 5:** Correlation between FSFI and ECRSHORT-FORMSCALE.

FSFΙ		Desire	Arousal	Lubrication	Orgarm	Satisfaction	Pain	Τotal Score
ECRSHORT-FORMSCALE	Anxiety	−0.389 **	−0.423 **	−0.254 *	−0.278 *	−0.267 *	−0.100	−0.334 **
	Avoidance	0.099	0.167	0.295 *	0.226	0.078	0.121	0.194
	Discomfort with closeness	−0.271 *	−0.250	−0.067	−0.109	−0.197	−0.289 *	−0.210

FSFI: Female Sexual Function Index; ECRSHORT-FORMSCALE: Experience in Close Relationships Scale. All indices report Spearman’s correlation coefficient, * *p* < 0.05, ** *p* < 0.005.

## Data Availability

Data are available upon reasonable request due to privacy, ethical, and legal restrictions associated with human subject questionnaire data.
